# ARAS recent onset acute phase psychosis survey, a prospective observational cohort of first episode psychosis in Iran—the cohort profile

**DOI:** 10.1038/s41537-022-00295-z

**Published:** 2022-11-19

**Authors:** Sara Farhang, Maryam Shirzadi, Rosa Alikhani, ARAS investigators, Shahrokh Amiri, Arash Mohagheghi, Reza Naghdi-sadeh, Ayyoub Malek, Alireza Shafiei-kandjani, Fatemeh Ranjbar, Ali Fakhari, Gholamreza Noorazar, Sepideh Herizchi, Golnaz Adalatzadeh, Anis Naderi, Behrooz Z. Alizadeh, Richard Bruggeman, Wim Veling

**Affiliations:** 1 Rob Giel Research Center, University Center for Psychiatry, University Medical Center Groningen, University of Groningen, Groningen, The Netherlands; 2 Research Center of Psychiatry and Behavioral Sciences, Tabriz University of Medical Sciences, Tabriz, Iran; 3 Clinical Research Development Center, Imam Reza Hospital, Kermanshah University of Medical Sciences, Kermanshah, Iran; 4 Department of Psychiatry, Psychosis Research Center, University of Social Welfare and Rehabilitation, Tehran, Iran; 5 Department of Epidemiology, University Medical Center Groningen, University of Groningen, Groningen, The Netherlands; 6 Department of Psychiatry, University Medical Center Groningen, University of Groningen, Groningen, The Netherlands; 7 Working group of psychiatry and psychology culture-based knowledge development, Tabriz University of Medical Sciences, Tabriz, Iran

**Keywords:** Schizophrenia, Psychosis

## Abstract

The Middle East is underrepresented in psychosis research. The ARAS recent onset acute phase psychosis survey (ARAS) is a longitudinal cohort across multiple centers in Iran, established to investigate characteristics, determinants and early course of psychosis in a non-Western, Middle East context. Here, baseline characteristics of the ARAS cohort are reported. The ARAS cohort enrolled patients with recent onset psychosis from September 2018 to September 2021 in East Azerbaijan, Kermanshah and Tehran, including Iranian patients from different sociocultural contexts. The baseline assessment included demographics, socioeconomic status, clinical (positive, negative, depressive symptoms) and psychosocial (religiosity, social support, self-stigma) characteristics, cognitive functioning, metabolic profile, substance use and medication use measured by validated questionnaires. These assessments will be followed up after one and five years. A total of 500 patients with a first episode of psychosis were enrolled from three provinces in Iran. With 74.1% being male, the mean age (SD) of patients was 32.3 (9.7) years. Nearly a quarter of patients was diagnosed with schizophrenia and 36.8% with substance induced psychotic disorder. Amphetamine (24%) and opium (12%) use were common, cannabis use was not (5%). Only 6.1% of patients lived alone while 29% of patients was married and had children. The majority of them had achieved secondary educational level and 34% had a paid job. The most common antipsychotic treatment was risperidone. There was a wide range for scores of PANSS, with 9.4% having dominant negative symptoms. The most common prescribed medication was risperidone. Near to 40% of patients had noticeable signs of depression and prevalence of metabolic syndrome was 13.4%. The majority of patients (57.2%) had moderate and 5.4% reported to have severe disability. More than 30% reported to be highly religious. Patients had the highest satisfaction with people living with, and the lowest for finance and job.

## Background

The majority of available research data about psychotic disorders originate from high-income countries^1^ which severely limits our understanding of the etiology, phenomenology, treatment and course of psychotic disorders. Differences between these mostly North American and European communities with those who have been less studied for psychotic disorders are not limited to the context of economic categorization, as there are also notable social, cultural, genetic and environmental discrepancies with populations in other regions of the world.

The Middle East is underrepresented in the psychosis literature. Mental health research in this region is growing in quality and scope, but there is a knowledge gap about schizophrenia spectrum (SSD) and other psychotic disorders in this region. There have been sporadic studies in the large population living in this region (including Iran) so far^2^ but there has not been a single psychosis cohort study to make a comprehensive conclusion. History of medicine in Iran is nurtured with acceptance of what is known as psychiatric symptoms nowadays, as symptoms of “*mental illness*”, acknowledgment of individuals with symptoms as “*patients*” as well as the initiation of treatment and care for patients in a medical setting, using herbal remedies and counselling^3^. Establishment of the first educational mental hospitals in 1946, probably made the most dramatic change to the mental health care system for patients in Iran. Quality and extent of health care system in Iran continued to develop, along with improvement of medical education^4^. Implementation of the up-to-date psychiatric knowledge to the practice was further achieved by the integration of mental health into the inclusive primary health care system in the country^5^.

These achievements raised questions toward a better understanding of patients with psychosis and their needs ranging from financial supports and available facilities, to diagnostic assessments and better treatments. Several studies have been published about epidemiology of psychiatric disorders in Iran, providing preliminary data about psychotic disorders^6^. While there are still no reliable rates of prevalence and incidence of psychosis, these reports suggest differences between Iranian and Western contexts in terms of risk and resilience factors related to psychosis. A good example is that opioids are the most common type of drugs used in Iran^7^, setting Iran apart from many other countries. Recently, there has been more interest towards pathways of care for Iranian patients with psychosis, and reports are provided that evaluate patients in different stages of untreated psychosis^8^, receiving treatment^9^ and aftercare^10^. They also show that not everybody has the same access to the mental health care services in academic centers in Iran, which are comparable to facilities in more developed counties^11^. Living in rural area or having a low income, limits access to these medical centers.

### Aims

The ARAS recent onset acute phase psychosis survey (ARAS)^12^ is an observational prospective cohort across multiple centers in Iran, established to fill knowledge gaps and provide a strong base for psychosis research and treatment, not only for Iran, but contributing to global mental health. Moreover, the study provides unique information about specific profile of risk and resilience factors different to those available in studies from other parts of the world. This will give a comprehensive view of clinical and social predictors of patient prognosis and lead to build a patient-centered management of psychosis. Registered patients will be invited for follow up, one, three and five years later. In this paper, we describe the baseline characteristics of the three-year baseline cohort.

## Materials and methods

This prospective cohort of the first episode psychosis (FEP) was designed by a steering committee from Department of Psychiatry, Tabriz University of Medical Sciences, Iran and Departments of Psychiatry and Epidemiology, University Medical Center Groningen, the Netherlands. The study protocol which is described in a published paper^12^, was approved by the Iranian national ethical committee (IR.NIMAD.REC.1396.101), and is funded by Iranian National Institute for research in medical sciences.

Patients were included only after giving written informed consent. by patients and their first-degree relatives or legal representative. As the ultimate goal of this cohort, is to establish a system for providing the best care for patients and their families, cooperation and willingness of both was valuable. There was no case in which there was incongruency between their decisions. Patients were aware that they could leave the study at any time for any reason with no influence on their care. A code was given to each patient and all data were recorded anonymously.

### Study population

**Fig. 1:**
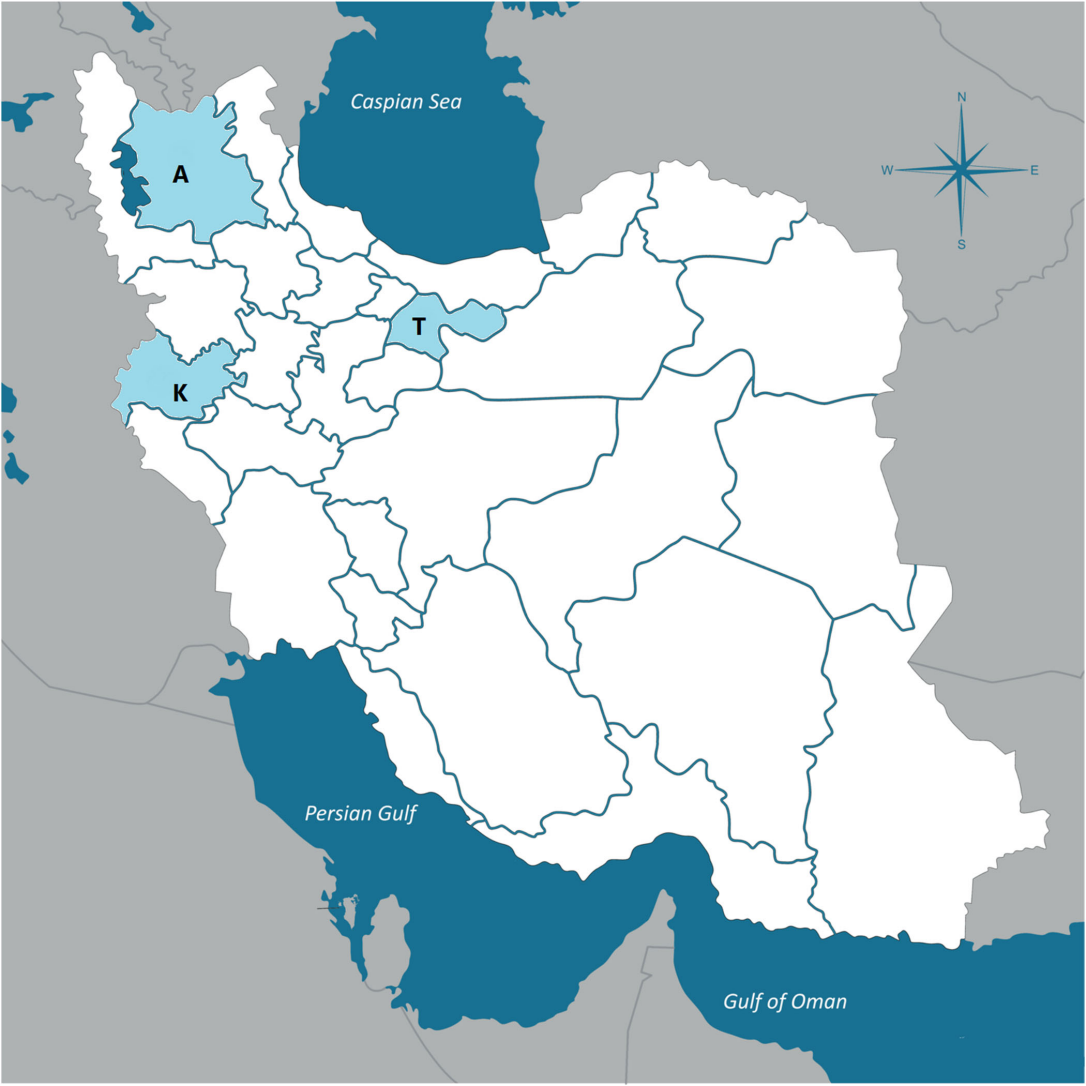
ARAS Recent onset Acute phase pSychosis Survey catchment area. Map of Iran, showing the three collaborating centers in ARAS cohort as east Azerbaijan (A), Tehran (T) and Kermanshah (K).

The ARAS cohort started in late 2018 with enrolling patients in East Azerbaijan, a province in the North of Iran, where the majority of inhabitants have Azeri ethnic background. After establishment of the research infrastructure, Kermanshah university of medical sciences, in Kermanshah province, and University of Social Welfare and Rehabilitations Sciences, located in Tehran, joined ARAS study. Kermanshah province is located in the west part of Iran and the Iraqi border. The majority of people living in this region is Kurdish. Tehran is the capital city of Iran, and the most populous city of western Asia. This destination for mass migrations is located in central part of the country, with a population consisting of Farsi speaking, Azeri, Lor, Kurd and other ethnicities (in that order). Thus, a sample of patients from three different contexts were included in the cohort (Fig. 1).

The Ministry of Health and Medical Education in Iran organizes the health care system through medical universities located in each province. The primary health care system and access to specialists throughout each province are organized by the referring system of the responsible medical university, that is in close contact with private sector as well. Although the exact rate of admission in the first episode of psychosis is not confirmed, it is common practice that the majority of patients with first episode psychosis are referred to academic hospitals and get admitted not only for treatment but also for a thorough diagnostic evaluation.

### Eligibility criteria

The study target population includes participants with (signs of) a recent onset (less than two years) psychotic disorder. The cohort includes patients with a clinical diagnosis of schizophrenia, schizophreniform disorder, delusional disorder, brief psychotic disorder, schizotypal personality disorder, schizoaffective disorder, or substance-induced psychotic disorder based on the Diagnostic and Statistical Manual of Mental Disorders (DSM)-5. All of the patients receiving any of these clinical diagnoses, who had their first sign or symptoms within the two years prior to the inclusion date, were invited to participate by the treating psychiatrist. Only those diagnosed with affective psychosis or psychotic symptoms due to other medical conditions were excluded.

### Measures

After being referred by their clinician, the study was explained to the patients and their caregivers and those who gave written consent were included. Test psychologists and psychiatrists conducting the assessments were trained by repeated workshops. Inter-rater concordance was examined for observer-rated tools by Kappa statistics. Measurement instruments are listed in Table 1, some were already available in Farsi language, and a few were translated for this project^12^.

**Table 1:** Process of assessments, applied instruments, and data collection process in ARAS.

Clinical symptoms	Structured Clinical Interview for DSM-5 Positive and Negative Syndrome Scale Calgary Depression Scale in Schizophrenia WHO Disability Assessment Schedule Global Assessment of Functioning Subjective Response to Antipsychotics-34
Metabolic profile	Laboratory testing Anthropometrics Pulse rate and blood pressure
Psychosocial profile	Manchester Short Assessment of Quality of Life Internalized Stigma of Mental Illness scale Multidimensional Scale of Perceived Social Support Stark and Glock’s dimensions of religiosity
Cognitive profile	Forward digit span task, Backward digit span task, Letter-number sequencing task Trail making task Auditory verbal learning test, Rey Osterrieth complex figure Symbol digit modality task, letter digit modality task, Verbal fluency task Stroop task Wechsler intelligence scale IV Benton Facial Recognition Test

#### Clinical characteristics

Evaluations started with Structured Clinical Interview for DSM-5 (SCID) or^13^ or the Kiddie-Schedule for Affective Disorders and Schizophrenia Present and Lifetime Versions (K-SADS-PL)^14^. The gathered information was used to score the severity of symptoms by the symptom severity dimension tool of DSM-5^15^, and Positive and Negative Syndrome Scale (PANSS)^16^. The Calgary Depression Scale in Schizophrenia^17^ was used to measure the severity of depressive symptoms. Medications, their side effects and the patients’ adherence were assessed using the Subject’s Response to Antipsychotics (SRA-34)^18^.

Assessment of functioning was done by WHO Disability Assessment Schedule (WHODAS 2.0)^19^, and Global Assessment of Functioning (GAF) Scale form DSM^20^ Their quality of life was evaluated by the Manchester Short Assessment of Quality of Life (MANSA)^21^.

#### Metabolic profile

After a physical examination, blood pressure, heart rate, height, weight, and waist circumference were measured. Laboratory tests for general health conditions, included lipid profiles, fasting blood sugar and glycohemoglobin A1c (HbA1c), liver function tests, and urine analysis for drugs.

Tabriz study center takes additionally blood samples to isolate whole blood, red blood cells, buffy coat, plasma and serum. Samples are stored in freezers at −80 °C available at the site until further analyses.

#### Cognitive profile

The neurocognitive battery included fifteen tasks covering MATRICS domains^22^. Working memory was tested by the forward and backward digit span, letter-number sequencing tasks and the Stroop test^23^. Divided attention was scored using the comprehensive test of the Trail making task. Memory was tested using the Rey Auditory Verbal Learning Test and the Rey-Osterrieth Complex Figure^24^. Executive functioning was evaluated using the symbol digit modality task, letter digit modality task, backward digit span, and letter-number sequencing task. Benton facial recognition task was used to give an estimation of social cognition^25^.

#### Psychosocial profile

Perceived support of the patients was scored by the Multidimensional Scale of Perceived Social Support^26^. An adapted version of the Stark and Glock’s dimensions of religiosity to the Islamic religion was used as a measure of religiosity^27^. The Internalized Stigma of Mental Illness scale (ISMI), gave an estimation of stigma in the study sample^28^.

Psychometric properties of these mentioned instruments are previously described in detail^12^.

#### Data management

Paper records are coded and restored for quality check. All of the data are stored in a web-based platform. Principal investigators from each center have access to data of the same center. The principal investigator of the leading center has access to all data and performed the quality check.

Quality control is performed at several levels. Raw data are checked automatically for invalid or nonsense entries when uploaded to the web-based platform. Recorded data are also compared to paper records by a third psychologist in each center. Results of the tools for each patient, are reviewed regularly by the principal investigator in each center, as well as the principal investigator of the leading center for concordance between data for each patient. In case of disagreement between results of different tools, or data recorded for separate variables, paper records are reviewed first, and then a third opinion was taken from a non-investigator psychiatrist.

## Results

The total number of referred patients were 578. From those who were referred to the study site for evaluations, 78 were excluded (not meeting the inclusion criteria = 63, no show = 15, eligible but refused to participate = 0, inability to cooperate with all tasks = 0). Finally, 500 patients were included with a first episode of psychosis from three provinces in Iran, between September 2018 and September 2021. More than 70% were male. Female patients included five pregnant and seven breast-feeding mothers.

The majority of patients were admitted to the hospital as inpatients and 7.2% of the included patients were enrolled in outpatient clinics. From those who were referred to the study site for evaluations, 15 did not attend which all were enrolled from the outpatient clinics. Their demographics are described in Table 2, specified by collaborating center. Some differences were observed between the three centers. Patients enrolled in Tabriz were younger (mean age 29.7 ± 11.2), than Kermanshah and Tehran, probably because of active collaboration child-adolescent psychiatrists of Tabriz, as well as older age of patients with substance induced psychosis which comprised higher ratio in Kermanshah center. A gender discrepancy is also noticeable between centers, with a higher rate of male patients enrolled in Tehran study center that happened due to religious rules, not allowing a male rater to visit female inpatient clinics in this center.

**Table 2:** Demographic characteristics of included patient in the ARAS cohort by the three study centers.

	Tabriz (*n* = 196)		Kermanshah (*n* = 149)		Tehran (*n* = 155)		Total (*n* = 500)		*p*
	Mean/%	SD/N	Mean/%	SD/N	Mean/%	SD/N	Mean/%	SD/N	
Age	29.7	11.2	33.4	9.3	36.3	10.4	32.3	9.7	0.004
Gender (% male)	62.8	123	76.5	114	85.8	133	74.0	370	0.005
Ethnicity									
Azeri	94.4	185	1.3	2	16.8	26	42.6	213	0.005
Kurd	5.1	10	85.2	127	5.8	9	29.2	146	
Fars	0.5	1	6.7	10	62.6	97	21.6	108	
Lor	0	0	1.3	2	9.7	15	3.4	17	
Other	0	0	5.4	8	5.2	8	3.2	16	
Marital status									
Single	60.7	119	48.3	72	60.6	94	57.0	285	0.05
Married	26.0	51	38.3	57	23.9	37	29.0	145	
Separated	2.0	4	1.3	2	13.5	21	5.4	27	
Divorced	9.7	19	11.4	17	1.3	2	7.6	38	
Widowed	1.5	3	0.7	1	0.6	1	1.0	5	
Number of children									
0	11	14.5	24	31.1	14	22.9	49	22.2	0.338
1	27	35.5	18	23.3	19	31.4	64	30.1	
2	25	32.9	23	29.8	21	34.4	69	31.8	
3 or more	13	17.1	12	15.9	7	11.4	32	15.9	
Living situation									
Original Family	66.3	130	57.0	85	78.1	121	67.2	336	0.998
Spouse/children	27.0	53	36.2	54	3.2	5	22.4	112	
Alone	6.1	12	5.4	8	7.1	11	6.2	31	
Other	0.5	1	1.4	2	11.6	18	4.2	21	
Educational level									
Lower	38.8	76	50.	75	36.1	56	43.6	207	0.058
Middle	50.3	90	35.6	53	52.9	82	45.8	225	
Higher	22.6	30	14.1	21	10.9	17	13.4	68	
Day time activity									
Paid job	24.1	47	47.6	71	53.5	83	40.2	201	0.005
Student	16.3	32	1.3	2	2.5	4	7.6	38	
No job	33.2	65	36.9	55	42.6	66	37.2	186	
Housewife	26.5	52	14.1	21	1.3	2	15.0	75	
Socioeconomical level									
Low	82.1	161	76.5	114	69.0	107	76.4	382	0.005
Middle	3.1	6	16.8	24	18.1	28	11.6	58	
High	14.8	29	7.3	11	12.9	20	12.0	60	

### Clinical characteristics

The primary psychiatric diagnosis is described in Table 3. Substance induced psychotic disorder and schizophrenia were the most common. The mood state of those with schizoaffective disorder was depressive in 3 (10.7%). The mean duration of untreated psychosis was 7.8 ± 7.9 months, with a wide range of less than one month to two years.

**Table 3:** The main diagnosis in the study population, by age group.

Main diagnosis	under 18	18–20	20–25	25–30	30–35	35–40	40–45	45–50	50–55	55–60	over 60	Total
Schizophrenia	17	5	19	24	15	11	6	8	4	1	0	110
Schizophreniform	0	2	9	5	2	6	3	1	1	0	0	29
Schizoaffective	2	0	1	5	6	5	3	3	1	1	1	28
Delusional disorder	2	4	5	8	9	12	5	4	2	5	2	58
Brief psychotic disorder	6	2	2	1	1	0	1	2	0	0	0	15
Other psychotic disorder	9	4	13	13	13	6	10	5	1	2	0	76
Substance induced psychotic disorder	1	6	18	39	35	39	22	12	7	3	2	184

Age distribution of the patients is shown in the same table. Patients with substance induced psychosis were older than those with no history of substance use. The most common substance used by patients was opium (Table 4), though the most common substance leading to the diagnosis of substance induced psychotic disorder was amphetamine. Cigarette smoking was quite common, but very few patients (about 5%) used cannabis. Polysubstance use (excluding nicotine) was reported in 18.2% of those who were current user.

**Table 4:** Substance use in patients included in ARAs cohort.

	Current use	History of use
Nicotine	162 (32.4%)	116 (23.2%)
Amphethamine	119 (23.8%)	58 (11.6%)
Opium*	91 (18.2%)	55 (11.0%)
Heroin**	59 (11.8%)	315(6.3%)
Alcohol	44 (8.9%)	78 (15.6%)
Cannabis	28 (5.7%)	31 (6.1%)
Other opioids	34 (6.8%)	39 (7.8%)

*preparations of the opium plant.

**Ten patients were intravenous drug users.

While 14.1% of patients was taking no medication, the majority of them were receiving antipsychotic treatment at the time of assessment: risperidone (29.4%), olanzapine (21.4%), clozapine (1.6%), quetiapine (19.7%) and aripiprazole (2.4%). First generation medication was prescribed for 11.4% (haloperidol 6.3%, perphenazine 1.1%, trifluoperazine 0.5, chlorpromazine 1.9%, fluphenazine 0.8, flupentixol 0.8). Increased appetite and dry mouth were the most common side-effects reported by patients, and the most common subjective benefits reported were decreased anxiety, and hearing less voices in those who had hallucinations. The mean duration of taking medication was 0.9 ± 2.1 months.

Patients were evaluated for different aspects of their clinical symptoms. Severity of symptoms as PANSS scores are shown in Table 5. The composite scale of PANSS showed that only 9.4% had dominant negative symptoms. Very few patients were already diagnosed to have depression by their treating psychiatrist, but 39.4% scored higher than six in the Calgary rating scale for depression. The majority of patients (57.2%) had moderate and 5.4% reported to have severe disability by WHODAS questionnaire. Results of MANSA indicated that patients had the highest scores for items evaluating satisfaction with people living with, health and sex life and the lowest for finance and job. About 50% of patients had GAF score of 21 to 30.

**Table 5:** Results of the baseline measurements in ARAS study.

	Instrument	Mean ± SD OR percentage	Range	Availability of data (%)
Clinical symptoms				
Symptom severity	Positive and Negative Syndrome Scale			100
	Positive scale	17.7 ± 5.1	7–32	
	Negative scale	12.4 ± 4.9	7–38	
	General psychopathology	26.7 ± 9.4	16–63	
Depressive symptoms	Calgary Depression Scale in Schizophrenia	5.7 ± 4.5	0–19	100
Medication (side) effect	Subjective Response to Antipsychotics-34	49.84 ± 6.0	36–68	95.8
Disability	WHO Disability Assessment Schedule	75.83 ± 19.76	33–136	100
Quality of life	Manchester Short Assessment of Quality of Life	46.1 ± 10.1	16–105	95.8
Social, occupational, and psychological functioning	Global Assessment of Functioning	28.5 ± 10.1	0–72	100
Metabolic profile				
Body mass index	= <18.5	9.7	13.6–44.9	100
	18.6_25	59.0		
	25.1_30	25.9		
	30.1_35	4.5		
	35<	1.0		
Components of metabolic syndrome	Fasting blood sugar (md/dL)	91.6 ± 19.5	59–282	100
	Blood pressure (mmHg)	110/70*	100/60–140/90	96.4
	Waist circumference (cm)	85.5 ± 12.4	48–125	96.4
	Triglyceride (md/dL)	11.3 ± 60.2	40–452	100
	Cholesterol (md/dL)	158.2 ± 42.1	29–331	100
Fulfilling metabolic syndrome		67 (13.4%)		
Cognitive performance				
Working memory	Letter-number sequencing task	3.6 ± 3.6	0–39	86.2
	Forward digit span task	7.2 ± 2.1	0–15	
	Backward digit span task	4.9 ± 2.0	0–11	
	Stroop task	5.2 ± 7.2	0–40	
	Stroop task (interference)	47.54 ± 260.7	−405–990	
Attention	Trail making task	79.9 ± 58.3	0–490	
Visual and auditory memory	learning	33.6 ± 12.5	0–65	84.4
	Word span	4.5 ± 2.0	0–12	
	recognition	6.34 ± 4.4	−30–36	
	Delayed recall	6.0 ± 3.3	0–15	
	Rey Osterrieth complex figure	7.9 ± 9.2	0–36	
Executive functioning	Symbol digit modality task	21.5 ± 13.1	0–57	86.2
	letter digit modality task	22.4 ± 13.3	0–65	
	Verbal fluency task	21.9 ± 11.5	0–52	
Social cognition	Benton Facial Recognition Test	16.1 ± 4.7	1–34	68.8
Psychosocial profile				
Social support	Multidimensional Scale of Perceived Social Support	43.89 ± 13.32	0–77	85.8
Religiosity	Stark and Glock’s dimensions of religiosity	75.23 ± 11.9	42–107	68.8
Stigma	Internalized stigma in mental illness scale	65.5 ± 11.6	32–104	83.8

*Mode

### Psychosocial profile

As described in Table 2, 31.2% of adult patients were married and one under 18 married female patient was recorded. We found no difference for the living status across the study centers, while marital status was different (*P* = 0.05) being the highest frequency of being single (60.7%) in Tabriz, being separated in Tehran (13.4%), and being divorced in Kermanshah (11.4%). Number of children was not different in the three centers and 76.7% of those who were not single had children. Except for 10% who lived alone or in supported environments, most of patients lived with their families.

Higher number of child and adolescent patients were enrolled in Tabriz, resulting in a higher rate of students at this center. As described before, very few female patients were recorded in Tehran, so the rate of housewives is lower at this center compared to the other centers. Having a paid job was less frequent in Tabriz cohort and a total of 34% of patients had a paid job. This may explain the observed lower socio-economic status (SES) by this cohort (Table 2). In general majority of patients belong to low SES households.

Mean score of perceived support is shown in Table 5, with higher levels of support from family members. On the self-report religiosity scale, 30.7% reported to be highly religious, and the remaining reported to be moderately religious. Level of internalized stigma was not high as shown in Table 5.

### Cognitive profile

More than 80% of patients could cooperate to complete cognitive tasks. A wide range of performance was recorded from patients. The mean scores and the standard deviation are presented in Table 5.

### Metabolic profile

Components of metabolic syndrome are also presented in Table 5. In general, 59.0% were in the healthy weight range. Prevalence of metabolic syndrome is 13.4% in this sample. Only five were already diagnosed to have diabetes mellitus, and 4 patients were already diagnosed to have hypertension Table 5.

## Discussion

This study reported the baseline characteristics of ARAS study, as the first cohort of first episode psychosis in the middle east. This large naturalistic observational cohort of 500 cases provides the opportunity of having reliable data about a population with different socio-cultural context from most of the available studies. Study centers are chosen to include three main ethnic groups in Iran.

Increasing information about mental health aspects leads to the awareness about shortcoming of available research because of biased sampling^29^. Available data fails to adequately account for contextual differences that influence all aspects of psychotic disorders from onset and course to outcome and system of care. Emerging literature on psychosis epidemiology in non-Western settings have been established, but the number is still not much^30^. Lack of diversity and its consequences on our understanding of etiology, phenomenology, course of illness and effect of interventions, has recently been acknowledged more. In a recent article, Burkhard et al. explain the need for diversity in psychosis research with important examples. The fact that none of the studies cited in the meta-analyses of the most relevant socio-environmental factors associated with psychosis are conducted in the Middle East or Africa, and the source of the information for knowing cannabis as a casual factor for psychosis, being limited to industrialized countries are good examples^29^. There have been high quality initiatives recently established to extend our knowledge of psychotic disorders in diverse settings, such as the INTREPID study, investigating incidence and determinants of psychosis in India, Trinidad and Nigeria^31^. Results of ARAS study is expected to contribute toward this goal.

Baseline characteristics given in this report, contain several interesting data. There were several similarities between this cohort and previous studies, like clinical symptoms and the medications, but interesting differences were observed^32^. Longer duration of untreated psychosis compared to reports from European countries was noticeable^33^ though there was a wide range and several factors might influence it.

The observed rates of married patients and number of patients having children in this study are higher compared to the reports from other countries^29,34^. The general pattern of marriage rate in this population might explain this rate. National surveys indicate that two third of Iranian adult population are married. The latest reliable data reports age at the first marriage to be 23 for women and 27 for men in Iranian population^35^. This is widely influenced by place of residence and cultural factors too. This issue is of significant interest as child bearing offer the possibility of passing genetic susceptibility to the next generations, and further, being married and having children offer the opportunity of achieving more social support for patients, and on the other hand, an increased load of responsibility. Though higher level of support that patient reported from their family members shows the benefit of their life style. Low level of stigma in these patients with a FEP was also interesting that any change will be observed in follow up visits.

Another difference is the pattern of substance abuse, which is similar in the ARAS cohort compared to reports from the Iranian general population^36^ but is quite different compared to those of other countries^37^. Cannabis abuse is infrequent, while the most common substance that induced psychosis was amphetamine. A recent survey in the Iranian population reported a mean age of people using amphetamines of 35 ± 11 years^38^, that is compatible with the higher age of patients with substance induced psychosis in this study compared to other diagnoses. The higher age of psychosis induced by substance is replicated in other reports^39^, but the mean age of these patients was higher compared to similar reports from European countries^40^.

Higher rate for metabolic syndrome is a general concern in available literature about patients with severe mental illness including psychosis^41^. However, the prevalence of metabolic syndrome was 13.4% in this sample, which is lower than in the 22.8% rate reported from the Iranian general population in the same age^42^ and lower than reports from more developed countries^32^. Further effect of different antipsychotic medications (therapeutic and side effects) in this population, and any discrepancy with current knowledge will be revealed in follow up visits. Further studies can include data about dietary habits and physical activity of patients in details.

It was not feasible, nor intended, to report all of the gathered data in this report. But along with the unique origin of this study, another strength of this study this cohort is using the same tools that are used in other cohort studies and makes it possible to compare results^28^. The database includes data for several risk and resilience factors, as well as outcome measures in several domains that have already been known to have important influence on daily life of patients. These high-quality measurements covering all key domains of psychosis, provide a comprehensive evaluation of the exposome. Therefore, this first episode cohort will potentially be a great base for further research like gene-environment and follow-up studies.

This study had several limitations. Recruitment and retention for prospective cohort studies are always challenging. A possible weakness includes missing those who do not seek psychiatric help because of less problematic symptoms, higher levels of perceived stigma, or low access to care. Access to primary health care, (which now includes certain levels of mental health care) is very good in Iran^43^. We tried to include most of first episode patients by making a network of collaboration between primary health care service, university centers and private practice. Though those patients who don’t make contact with health care system in the first two years for any reason, are missed. Date of the first sign or symptom, that was the base for definition of FEP (as well as the duration of untreated psychosis) was reported by the patients and other sources of information and recall bias cannot be excluded. The majority of recruited patients were admitted to the hospital. Most Iranian psychiatrists prefer to admit patients with FEP to a hospital for thorough evaluations. Though not all of patients get admitted, especially those with a higher level of functioning, but the rate of rejection was not clear as we did not have an online referral system. The three contributing centers could enroll the majority of ethnicities in Iran, except for Arab and Baluch populations who live in Southern parts of Iran, representing 2–4% of the total Iranian population. Recently one of the important university hospitals in South of Iran has joined ARAS cohort and will address this limitation.

Beside learning from previous experiences, we were also flexible to handle the limitations of the covid-19 pandemic in part. The pandemic situation increased the rate of “no show”. Follow up visits will be online when patients prefer, that might influence the validity of measurements to some extent.

## Conclusion

ARAS study is the first observational cohort of the FEP in the Middle East region. This large representative Iranian cohort for the FEP, provides the opportunity of exploring the risk, resilience and genome-exposome interactions within this sociocultural context. Interesting differences and similarities are obvious in the baseline characteristics of this ample compared to reports from Western and more developed countries. The comprehensive evaluation can be the base for further research to fill the gap of knowledge about psychotic disorders.

## Supplementary information

Supplementary table
