# Supplementary material for: ARAS recent onset acute phase psychosis survey, a prospective observational cohort of first episode psychosis in Iran—the cohort profile
Source: Schizophrenia (Heidelb). 2022 Nov 19;8(1):101. doi: 10.1038/s41537-022-00295-z (PMC9675807; doi:10.1038/s41537-022-00295-z)
Supplement: Supplementary file 1 — Supplementary table [file 41537_2022_295_MOESM1_ESM.pdf]

Supplementary data: Score of PANSS in ARAS patients

	Mean	Standard deviation
Delusions	3.64	1.20
Conceptual disorganization	1.99	1.04
Hallucinations	2.93	1.35
Excitement	1.88	1.21
Grandiosity	1.96	1.22
Suspiciousness/persecution	3.14	1.24
Hostility	2.15	1.17
**Negative scale**		
Blunted affect	2.02	1.10
Emotional withdrawal	1.97	0.95
Poor rapport	1.79	0.95
Passive/apathetic social withdrawal	2.05	1.14
Difficulty in abstract thinking	2.28	1.11
Lack of spontaneity and flow of conversation	1.46	0.69
Stereotyped thinking	1.28	0.62
**General psychopathology**		
Somatic concern	1.38	0.76
Anxiety	1.84	1.02
Guilt feelings	1.75	0.96
Tension	1.44	0.72
Mannerisms and posturing	1.18	0.51
Depression	1.96	1.12
Motor retardation	1.48	0.74
Uncooperativeness	1.57	0.89
Unusual thought content	1.94	1.26
Disorientation	1.48	0.82
Poor attention	1.69	0.90
Lack of judgment and insight	2.51	1.29
Disturbance of volition	1.82	0.84
Poor impulse control	1.61	1.00
Preoccupation	1.46	0.77
Active social avoidance	1.92	1.11
